# Biomass Production from Electricity Using Ammonia as an Electron Carrier in a Reverse Microbial Fuel Cell

**DOI:** 10.1371/journal.pone.0044846

**Published:** 2012-09-19

**Authors:** Wendell O. Khunjar, Asli Sahin, Alan C. West, Kartik Chandran, Scott Banta

**Affiliations:** 1 Department of Earth and Environmental Engineering, Department of Chemical Engineering, Columbia University, New York, New York, New York, United States of America; 2 Department of Chemical Engineering, Columbia University, New York, New York, New York, United States of America; Texas A&M University, United States of America

## Abstract

The storage of renewable electrical energy within chemical bonds of biofuels and other chemicals is a route to decreasing petroleum usage. A critical challenge is the efficient transfer of electrons into a biological host that can covert this energy into high energy organic compounds. In this paper, we describe an approach whereby biomass is grown using energy obtained from a soluble mediator that is regenerated electrochemically. The net result is a separate-stage reverse microbial fuel cell (rMFC) that fixes CO_2_ into biomass using electrical energy. We selected ammonia as a low cost, abundant, safe, and soluble redox mediator that facilitated energy transfer to biomass. *Nitrosomonas europaea,* a chemolithoautotroph, was used as the biocatalyst due to its inherent capability to utilize ammonia as its sole energy source for growth. An electrochemical reactor was designed for the regeneration of ammonia from nitrite, and current efficiencies of 100% were achieved. Calculations indicated that overall bioproduction efficiency could approach 2.7±0.2% under optimal electrolysis conditions. The application of chemolithoautotrophy for industrial bioproduction has been largely unexplored, and results suggest that this and related rMFC platforms may enable biofuel and related biochemical production.

## Introduction

The production of biofuels and related chemicals from renewable resources has emerged as one of the great engineering challenges of the 21^st^ century [1]–[4]. The exploitation of photosynthetic organisms has been the predominant paradigm used for producing biomass, biofuels, and related chemicals. However, this approach is intrinsically limited by several factors including the low energy capture and transfer efficiency of photosynthesis [5]–[8]. Therefore there has been a great deal of interest in the development of alternative technologies that simultaneously improve energy capture and transfer to biosynthetic pathways optimized for production of useful compounds [9]–[14]. One way to address this challenge is through the development of reverse microbial fuel cells (rMFCs). In a standard microbial fuel cell, organisms oxidize organic fuels and transfer electrons into an electrochemical system so that fuels are converted to electrical energy. In a rMFC, this process is reversed so that electrical energy is used by cells to drive carbon dioxide fixation to high energy organics. The critical challenge for this approach is that energy must be efficiently transferred from an electrode into a biological host which is capable of using this energy for biosynthesis. rMFC platforms could have a significant impact in the biofuel and biochemical arena as they would be capable of using electricity generated from all renewable sources including wind, geothermal, hydroelectric, nuclear, and solar. They could be used in a variety of global locations, and they could be used for long term storage of excess electrochemical energy.

One approach to creating rMFCs is to use direct electron transfer from an electrode to the cells. This has been termed microbial electrosynthesis [15]. While this energy transfer can be accomplished directly, where electroactive cells in a biofilm utilize electrons from an electrode for anabolism [15], [16], diffusion issues and the requirement for 2-D biofilms make direct microbial electrosynthesis a challenging proposition.

An alternative approach to creating rMFCs is to use soluble electron mediators that can shuttle electrons from the electrode to the cells. The use of a mediator enables the utilization of planktonic cells in the bioreactor and facilitates easy 3-D scale-up of the individual components. Furthermore, the use of mediators can also enable separate-stage designs that afford spatial and temporal decoupling of energy capture and bioproduction. This can allow both processes to be operated and optimized separately. In the mediated approach, electrons are first transferred from an electrode to a soluble mediator and then the mediator would be oxidized by the cell. Inorganic compounds that are linked with chemoautotrophy and can be electrolytically regenerated, like hydrogen (H_2_) as well as those involved with the nitrogen, iron and sulfur biogeochemical cycles (i.e. ammonia (NH_3_), nitrite (NO_2_^−^), iron (Fe^2+^), hydrogen sulfide (H_2_S)) are attractive options for use in this platform since they can facilitate the construction of multi-carbon organics from carbon dioxide using naturally occurring carbon fixation pathways. These inorganic compounds naturally yield sufficient energy to support biomass growth and can be reduced via electrolysis (**Table S1**).

**Figure 1:**
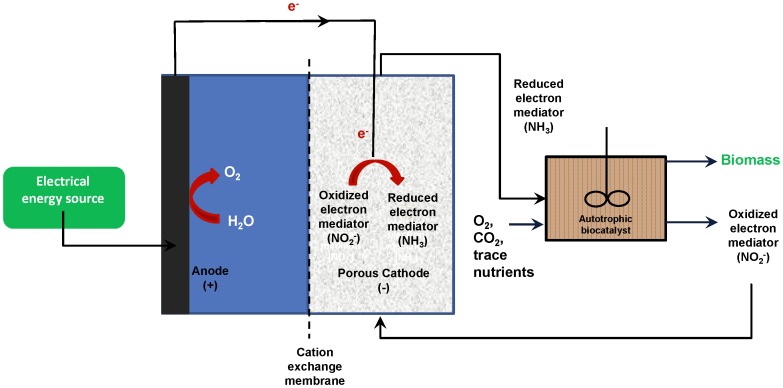
Overview of a reverse microbial fuel cell which uses the ammonia/nitrite redox couple as a mediator and ammonia-oxidizing *Nitrosomonas europaea* cells as biocatalysts.

Successful rMFC operation has recently been demonstrated using electrochemically produced formate coupled with genetically modified *Ralstonia eutropha* cells that were able to produce isobutanol [17]. In this version of the rMFC, carbon dioxide was electrochemically fixed into formate at the electrode, which was subsequently used by cells to produce a biofuel. In this paper, we address the feasibility of using an alternative electron transfer mediator and chemolithoautotrophy for primary production. We demonstrate sustained biomass production in a rMFC that has two components: 1) an electrochemical reactor that produces ammonia from nitrite using electrical energy, and 2) a biological reactor that containing a naturally chemolithoautotrophic, ammonia-oxidizing bacterium, *Nitrosomonas europaea* (**Figure 1**). This organism was selected as it is a well-studied autotrophic ammonia oxidizing bacterium whose genome has been sequenced [18]. We report stable long term operation (>15 days) of the separate-stage rMFC, which facilitated fixation of CO_2_ via the Calvin-Benson-Bassham (CBB) cycle using energy derived solely from ammonia. These results suggest that this approach can be expanded to produce biofuels and other chemicals via genetic modification of the *N. europaea* cells in the bioreactor without the need for photosynthesis.

## Results

Successful operation of a rMFC platform requires the selection of soluble mediators that not only support biosynthesis but can be efficiently recycled. In this study, we utilize the ammonia/nitrite couple as the soluble mediator. Consequently, experiments were designed to, 1) further our understanding ammonia regenerated via electrolysis and, 2) demonstrate that electrochemically derived ammonia can support biomass production. In confirming that biomass can be produced using this approach, it is our intention to showcase a flexible bioproduction strategy that could be expanded for the production of biofuels and biochemical via genetic engineering strategies.

### Electrochemical Reduction of Nitrite to Ammonia

**Figure 2:**
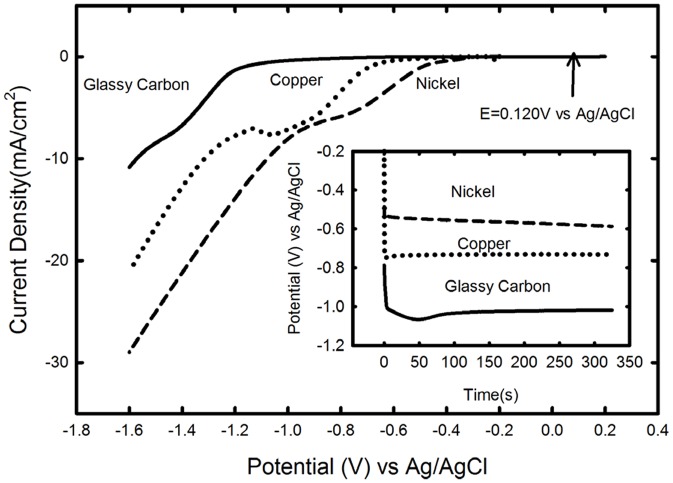
Linear sweeps of nickel, copper and glassy carbon rotating disk electrodes in 50 mM Phosphate Buffer +50 mM Sodium Nitrite at pH 7.0 at 400 **rpm.** Inset : constant current (2 mA/cm^2^) experiments in 50 mM Phosphate Buffer +50 mM Sodium Nitrite at pH 7.0 with nickel, copper and glassy carbon rotating disk electrodes at 400 rpm.

Since nitrogen can have oxidation numbers ranging from +5 to −3, the kinetics and selectivity of the nitrite reduction reaction are highly dependent on various criteria including the electrolytes, cathode materials, operating conditions [19]. In this work, copper, nickel and glassy carbon electrodes were screened as cathode materials for catalyzing the electrochemical reduction of nitrite to ammonia based on previous literature on nitrate/nitrite reduction. (E_pH 7.0_ = 0.12 V vs. Ag/AgCl). Copper electrodes were found to be highly active for nitrate reduction both in basic and weakly alkaline solutions [20], [21]. Nickel electrodes were chosen for their high selectivity to ammonia as the end product [20]. Glassy carbon was investigated as a cathode candidate because of its inertness in biological systems [22], [23]. Results from linear sweep experiments using a phosphate buffer solution (pH 7; 50 mM) supplemented with sodium nitrite (50 mM) indicated that the onset of nitrite reduction was different for each material (**Figure 2**). Constant current experiments (2 mA/cm^2^) indicated that the nickel cathode (0.65 V) had the lowest overpotential (additional potential required over thermodynamic potential) followed by copper (0.85 V) and glassy carbon (1.13 V). Even though glassy carbon displayed the highest overpotential among the three cathode materials tested, it was selected for further study due to its biocompatibility, inertness and long term performance [22]–[25].

### Electrochemical Regeneration of Ammonia from Spent Culture Media

**Figure 3:**
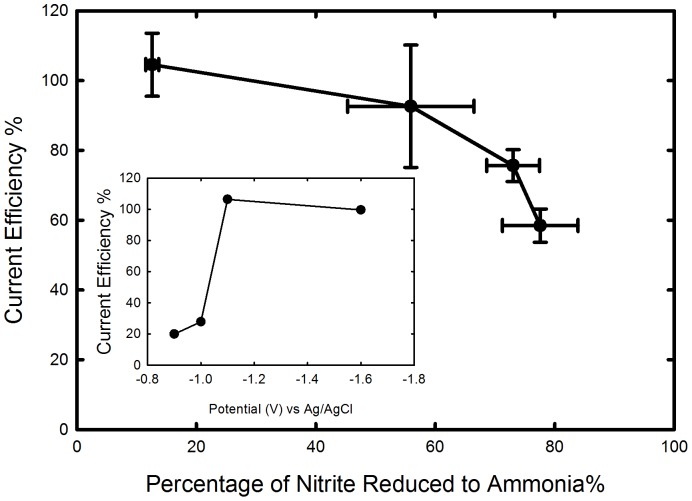
Current efficiency of nitrite reduction to ammonia as a function of percentage of nitrite converted to ammonia in spent media from the bioreactor. Inset shows the current efficiencies for 20 mM sodium nitrite in 100 mM phosphate buffer as a function of applied potential in batch experiments in an undivided three electrode cell with a glassy carbon cathode.

Further experiments investigated the feasibility of using glassy carbon cathodes to electrochemically produce ammonia from spent growth media obtained from *N. europaea* cultures. Initial current efficiencies (ratio of current for nitrite reduction to ammonia to overall current) of 104.5%±9.0 were observed in the presence of high nitrite concentration (50 mM). However, as the nitrite concentration decreased to 22% of the initial concentration (11 mM), the current efficiency decreased to 58.4%±4.7 (**Figure 3**). This is because mass-transfer limitations arise and there is insufficient nitrite flux to the electrode to allow the constant set current to result only in nitrite reduction. A second reaction thus increases in importance, and this reaction is believed from thermodynamic considerations to be the evolution of hydrogen gas. This decrease in current can be avoided by further optimizing the operating conditions at higher conversions. To demonstrate this latter point, constant applied potential experiments with an undivided three electrode cell set-up were performed using 20 mM nitrite in phosphate buffer. Near 100% current efficiency was obtained under optimal operating conditions with lower nitrite concentrations (**Figure 3** **inset**). These findings demonstrate that electrolysis is an effective approach for converting renewable electrical energy into chemical energy (NH_4_^+^/NH_3_).

### Performance of the Flow Cell Components

No signs of corrosion and electrode passivation at the anode and cathode or precipitation in the catholyte and anolyte were observed in experiments using spent *N. europaea* culture media. However, an increase in catholyte pH was initially observed. This pH increase was due to production of OH^−^ when nitrite is reduced to ammonia at the cathode and could not be offset by the proton content of the anolyte used in this experiment (phosphate buffer at pH 7.0). To prevent this increase, pH control of the catholyte via addition of hydrochloric acid (5 M) was performed. This resulted in ∼3% dilution of the catholyte. Alternatively, use of an acidic anolyte and a proton exchange membrane would compensate for OH^−^ produced at the cathode and would eliminate catholyte dilution in future systems.

### Operation of the Separate-stage Reverse Microbial Fuel Cell (rMFC)

**Figure 4:**
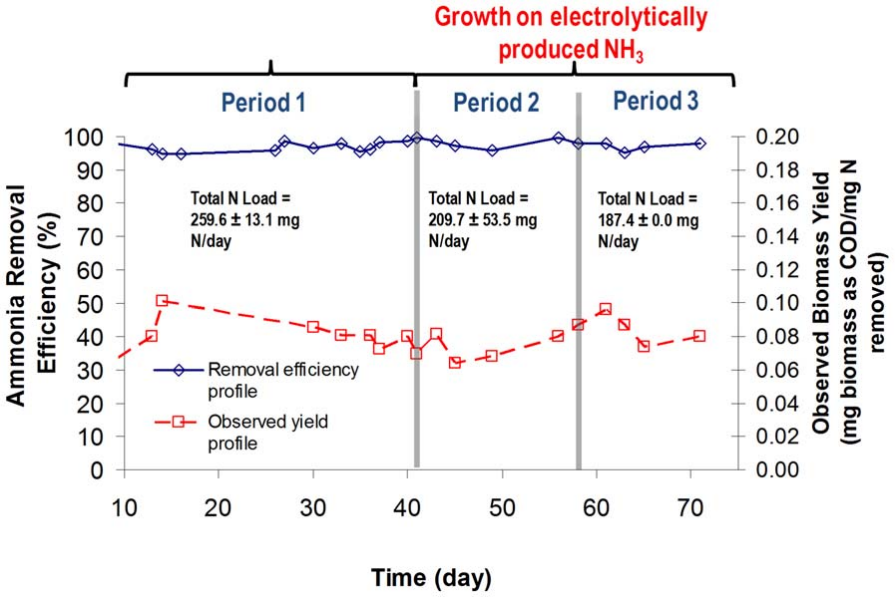
Ammonia removal efficiency and observed biomass yield for continuous flow cultivated *Nitrosomonas europaea*. Period 1 was defined by growth on synthetic media prepared from ACS grade chemicals. Period 2 was defined by cultivation on spent media from Period 1 that was electrochemically regenerated. Period 3 was defined by cultivation on spent media from Period 2 that was electrochemically regenerated. COD represents the chemical oxygen demand associated with complete oxidation to CO_2_. Replicate results are presented in Figure S1.

Once efficient electrochemical generation of ammonia from nitrite was demonstrated, experiments were performed to verify biomass growth using the regenerated ammonia. Results confirmed that this approach is a viable biosynthesis technology with electrochemically produced ammonia being successfully used to cultivate wild-type *N. europaea* (**Figure 4** **and Figure S1**) in replicate continuous flow reactors. Ammonia removal efficiency was maintained above 96% during all periods of operation (Period 1 (growth on synthetic media prepared from ACS grade chemicals) = 97.2±1.5%; Period 2 (growth on electrochemically generated ammonia from period 1 effluent) = 97.9±1.3%; Period 3 (growth on electrochemically generated ammonia from period 2 effluent) = 95.5±2.7%) while observed biomass yields during these experiments were statistically identical (Period 1 = 0.06±0.01 mg biomass/mg N; Period 2 = 0.06±0.02 mg biomass/mg N; Period 3 = 0.06±0.01 mg biomass/mg N; *P* = 0.99), demonstrating that the free energy efficiency of ammonia utilization for biomass construction (ratio of energy present as organic carbon divided by energy produced by ammonia oxidation to nitrite [26]) was steady (**Figure 1** **, Figure S1,** **Table 1** **and Table S2**). Biokinetic and stoichiometric parameters describing growth (maximum specific growth (µ_max_; n = 54), biomass yield coefficient (f_S_; n = 22), half saturation constant (K_NH3_; n = 22)) were also statistically unchanged across all culturing periods ((**Table 1** **and Table S2;** for µ_max,_ *P* = 0.11; For K_NH3_, *P* = 0.15; for f_S_, *P* = 0.48), and were within ranges previously reported by other researchers [27]–[32]. Collectively these results demonstrate that the application of electrochemically processed ammonia did not impact the aerobic metabolism of *N. europaea*. These finding are not unexpected since all nutrients (ammonia, oxygen, inorganic carbon, trace metals) were provided in excess during chemostat cultivation and during short-term assays.

**Table 1:** Summary of biokinetic and stoichiometric parameters describing growth of wild-type *Nitrosomonas europaea* on electrochemically regenerated media.

	Maximum specific growthrate (µ_max_;1/day)	Half saturation coefficient (K_NH3_;mg N/L)	Biomass yield coefficient (f_S_;mg biomass as oxygen equivalents/mg N)	Free energy efficiency (%)
Period 1^a^	0.50±0.15	2.51±0.65	0.06±0.01	4.4±1.6
Period 2^b^	0.36±0.07	2.57±0.63	0.05±0.02	5.1±1.0
Period 3^c^	0.24±0.08	3.19±1.34	0.05±0.02	4.9±0.6

a n = 8,

b n = 6,

c n = 8.

Replicate results presented in Table S2; mean ±95% confidence interval.

## Discussion

Coupling electrolysis and biosynthesis in a rMFC using mediated electron transfer requires an electron mediator that is inexpensive, highly soluble, non-flammable, and amenable to long term storage. In addition, this mediator must be compatible with the electrochemical cell with low power requirements for regeneration and it must also be capable of being efficiently utilized by the biocatalyst. Bacteria that have evolved to obtain energy from natural redox mediators will have optimized this process, and are likely ideal for use in rMFC systems. Five inorganic redox active compounds that are capable of supporting lithoautotrophy were considered for this purpose (NH_3_, NO_2_^−^, Fe^2+^, H_2_S, H_2_). From a thermodynamic perspective, the minimum power required for biomass production, which is equal to the Gibbs free energy of formation of the biomass, is equivalent for these five mediators; however, since the energy state of electrons varies per mediator, the rate of reduced mediator consumption and subsequent regeneration is governed by the biocatalyst ability to synthesize biomass using the energy from the mediator. This ability can be quantified by examining biomass growth characteristics such as specific growth rates and yield coefficients for each system. Comparison of biomass growth kinetics and yields associated with the five mediators indicates that H_2_, H_2_S and Fe^2+^ oxidizers can produce biomass more rapidly than ammonia and nitrite oxidizers (**Table S3**); however, the low water solubility, high flammability and toxic nature of H_2_ and H_2_S require complex storage and delivery capabilities, rendering use of H_2_ and H_2_S in rMFCs challenging (**Table S4**). Similarly, Fe^2+^ is unstable under aerobic conditions and will undergo rapid abiotic oxidation to Fe^3+^, making storage and sustained use of this mediator potentially difficult. In contrast, NH_3_ and NO_2_^−^ are highly water soluble, less toxic/flammable than H_2_S or H_2_ and can be stored under aerobic conditions. Additionally, these compounds are ubiquitous as environmental pollutants, rendering them inexpensive and suitable for use in chemolithoautotrophic bioreactors.

The power required for electrochemical regeneration of the chosen mediator (ammonia in this case) will be impacted by the cell overpotential. Using the experimentally determined overpotential (**Figure 2**) for glassy carbon (1.13 V) electrodes operating at 2 mA/cm^2^ and an overpotential of ∼0.400 V for oxygen evolution [33]_,_ and the thermodynamic cell potential of −0.497 V a cell potential of −2.0 V was calculated. (The lowest cell potential achieved experimentally in the flow-by cell was 4.0 V. This was predicted to be largely due to IR losses and can be minimized with improved design of the flow cell.) Using Faraday’s law and assuming 100% current efficiency, this suggests that a charge of 162 Ahr is required for the reduction of 1 mol of nitrite to ammonia, resulting in a power requirement of 0.324 kWhr (162 Ahr×2 V). Using the experimentally measured biomass yield of 0.06±0.01 mg biomass/mg N (**Table 1**), the power requirement for biomass production in this system was estimated to be 0.386 kWhr/g biomass (1.93 cents per g biomass assuming 0.05$kWhr).

In this study, the free energy efficiency of ammonia conversion to biomass was experimentally determined to be 4.81±0.37% (**Table 1**). This efficiency was calculated by computing the ratio between the total amount of energy yielded from ammonia oxidation to nitrite during cultivation (230 kJ/mol N) to the energy conserved in biomass produced in these experiments (305 kJ/mol biomass C) [26]. Assuming an energy capture efficiency of 56.5% during electrolysis (as calculated from overpotential experiments), we calculated that the overall energy efficiency of biomass production in this separate-stage rMFC system could approach 2.71±0.21%. This was calculated by multiplying the energy capture efficiency of electrolysis times the free energy efficiency of *N. europaea* growth. In these experiments, electricity generated from non-renewable sources was used; however, it should be noted that electricity derived from renewable sources can be easily substituted. In such a scenario, if one further assumes that the electrical energy used during electrolysis is derived from solar sources (conversion efficiency of 10% [34]) the biomass production efficiency of this ammonia-based rMFC system could be calculated by multiplying the conversion efficiency of solar capture times the overall energy efficiency of biomass production. In this study, the biomass production efficiency approached 0.27±0.02%. This level of efficiency is within the same order of magnitude as photon to biomass conversion efficiencies approaching 1% that is observed for contemporary photosynthetic systems [7]. It should be noted that while these calculations do not account for aeration and pumping energy requirements, they do take into account the energy requirement associated with electrolysis, which is expected to dominate the energy balance of this system. These results indicate that despite the high energy requirement for carbon fixation that is endemic to all lithoautotrophs, the current iteration of this rMFC platform can already yield high molecular weight organic material at a level that is comparable to the conventional photosynthetic paradigm.

Continuous operation of this ammonia based rMFC would need to resolve issues associated with nitrogen losses and inorganic carbon and proton supply. In the present study (2L scale), total nitrogen losses accounted for 5.4 mg N/day (2 mg N/day as biomass and ∼3.4 mg N/day as gaseous N oxides [35]). This nitrogen can be offset through the supplementation of exogenous ammonia obtained from municipal or industrial (e.g. animal feeding operations) sources or from ammonia purchased at the market value ($0.30±0.14/kg N based on 10 year average from 2000 to 2009 [36]). Similarly, inorganic carbon (IC) needs to be supplied to ensure sufficient substrate for biomass fixation (0.086 mol C per mol of nitrogen processed). Sodium bicarbonate/carbonate mineral ($0.09±0.03/kg CO_3_^2−^ based on 10 year average from 2000 to 2009 [36]) or CO_2_ enriched air ($0.02/kg CO_2_) [37] can be used to deliver this critical macronutrient. In this study, exogenous inorganic carbon (320 mg C/day) was added using a saturated sodium bicarbonate solution. Proton supplementation will also be needed to replenish those lost in the biomass purge stream.

A significant difference in the use of autotrophic cells in a rMFC as compared to heterotrophs will be decreased cellular growth rates (**Table S3)**. However, it is not clear if this is a significant limitation, as the important parameter in the development of rMFC platforms is the production rate of the final product of system. If the cells can be engineered to fix CO_2_ into chemicals or fuels with a cellular low growth rate, this may be beneficial as compared to faster growing heterotrophs. Of course, this can also be addressed through reactor design, where the hydraulic and solids retention times can be decoupled to increase production in the chemostat.

We recognize that neither electrolysis of nitrite to ammonia nor *N. europaea* growth on ammonia is novel; however, the coupling of both processes to produce biomass provides a significant proof of concept towards using mediated rMFCs as a viable strategy for producing organics from carbon dioxide using electrical energy. This approach can potentially be extended for direct production of high molecular weight organics, like aldehydes, alcohols, alkanes or alkenes directly from CO_2_ [11], [14]. To accomplish this, genetic modifications of lithoautotrophs aimed at directly producing and secreting high energy organics will be needed. This will enable cells to directly produce biochemicals or biofuels without the need for further processing as is required for the utilization of biomass obtained from photosynthesis. Obviously in these future application, the overall process efficiencies and yields will depend on the ability of the organism to channel energy and carbon away from biomass synthesis and towards biofuel and biochemical production pathways. Tolerance of produced toxicants could also constrain production. *In silico* simulations examining metabolic fluxes could provide insights into strategies for maximizing biosynthesis while minimizing unwanted cellular processes [38]–[40]. Thus, the efficiency of electrical energy conversion to biofuel using a similar approach would not be necessarily restricted to the efficiencies obtained with this proof-of-principle study. Collectively, these results show that although in its infancy, rMFCs offer great promise as an alternative to photosynthetic primary production. Future work will need to explore the use of alternative mediators and genetically modified biocatalysts for the purpose of generating biomass or biofuel products.

## Methods

### Strain and Growth Conditions

*Nitrosomonas europaea* ATCC 19718 was cultured in a chemostat (2 L; dilution rate = 0.2 day^−1^) utilizing modified autotrophic medium (designated SynM) comprising (mg/L): MgSO_4_·7H_2_O (200), CaCl_2_·2H_2_O (20), K_2_HPO_4_ (87), KH_2_PO_4_ (8820), EPPS (2520) Na_2_MoO_4_·2H_2_O (0.01), MnSO_4_·H_2_O (0.017), CoCl_2_·7H_2_0 (.0004), CuCl_2_·2H_2_O (0.17), ZnSO_4_·7H_2_O (0.01), chelated iron (1), NaNO_2_ (690) and (NH_4_)_2_SO_4_ (2,640) [41]. Sterile aeration was provided via air pumps fitted with 0.22 µm HEPA® filters (gas flow rate = 7.5 L/min). pH (7.5±0.01) was controlled via automated addition of NaHCO_3_ (80 g/L). Culture purity was confirmed via direct cell counts and DNA sequencing [42].

### Growth Experiments with Electrochemically Produced Media

Upon achieving steady state removal of ammonia (defined as three consecutive days with an effluent NH_3_ concentration that was not statistically different at the 95% confidence interval from the three previous time-points; defined as Period 1), the autotrophic media described previously was replaced with spent media (from Period 1) that was electrochemically regenerated once or twice as described below (defined as Periods 2 and 3 respectively). Reactor performance was then monitored for changes in cell counts, nitrogen species (ammonia, nitrite and nitrate), biokinetic and stoichiometric parameters. Statistical comparison of parameter estimates (yield coefficients, growth rates and half saturation constants) from Periods 1 through 3 was performed using univariate analysis of variance (ANOVA). The significance level (α) used was 0.05 and the corresponding *P*-values (measure of consistency among experimental values) are reported where appropriate.

Nitrogen mass balances were constructed from running averages of ammonia, nitrite and cell count profiles, before during and after experiments to account for the changes in overall nitrogen loading. Carbon mass balances were also constructed by estimating the mass of CO_2_ transferred from aeration using a 2 film model to estimate K_LA,CO2_from the measured oxygen mass transfer coefficient (K_LA,O2_ = 0.0054 1/s) [43], and the mass of bicarbonate added for pH control as well as carbon lost from cell purge. All experiments were conducted in duplicate.

### Biomass Activity Measurements

Oxygen uptake rates (OUR) were determined in duplicate for chemostat cultured *N. europaea* cells using an YSI model 5331 oxygen electrodes (YSI Incorporated, Yellow Springs, OH) connected to a dual channel meter (model 5300A; YSI Incorporated, Yellow Springs, OH) such that DO readings were recorded every 10 seconds using an automated data acquisition system (Labview 8.0). Specific OUR (sOUR_max_) values were determined by dividing the OUR values by the cell concentration of each sample. Yield coefficients, growth rates and half saturation constants were estimated using extant batch procedures described elsewhere [44].
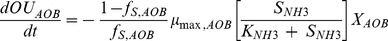
(1)


where

OU_AOB_ = active oxygen uptake due to ammonia oxidation to nitrite (mg/L)

µ_max_,_AOB_ = maximum specific growth rate of *Nitrosomonas europaea* (1/day),

f_S,AOB_ = yield coefficient for *Nitrosomonas europaea* (mg biomass/mg NH_3_-NOD)

S_NH3_ = ammonia concentration (mg NH_3_-NOD/L),

X_AOB_ = biomass concentration (mg biomass as COD/L),

K_NH3_ = half saturation constant (mg NH_3_-NOD/L).

NOD = nitrogenous oxygen demand (1 mg NH_3_ = 3.43 mg NOD when ammonia oxidation to nitrite is considered)Wet Chemistry and Analytical Chemistry

Cell counts *w*ere performed using a Brightline hemocytometer (Hausser Scientific, Horsham PA). Samples for nitrogen analyses were filtered through 0.45 µm filters prior to immediate analyses for nitrite (Griess reagent) and ammonia (gas-sensing electrode, ThermoScientific, Waltham MA) [45]. Trace metal analyses (Mg, Ca, Fe, Cu) were performed on acidified (2% HNO_3_) filtered samples (0.45 µm) using inductive coupled plasma atomic emission spectroscopy (Horiba ACTIVA-M ICP-AES, Edison NJ).

### Linear Sweep Voltammetry

Linear sweep voltammetry experiments were conducted using a µAutolab Potentiostat (Ecochemie, Netherlands). Glassy carbon rotating disk electrodes (3 mm diameter) were produced in-house as described in Gallaway et al [46]. Nickel and copper were electrochemically deposited on a 4 mm diameter platinum rotating disk electrode. An Ag/AgCl reference electrode and a platinum wire counter electrode were also used. A sweep rate of 5 mV/s was used to scan between 0.2 to −1.6 V versus Ag/AgCl.

### Rotating Disk Electrode Constant Current Experiments

A three electrode cell described above consisting of a Ag/AgCl reference electrode, a platinum counter electrode and a nickel, glassy carbon or copper rotating disk electrode was also used in constant current experiments. The electrode was rotated at 400 rpm and constant current of 2 mA/cm^2^ was applied for 325 s using µAutolab Potentiostat (Ecochemie, Netherlands).

### Constant Potential Experiments

A three electrode cell similar to the one described above was used in constant applied potential experiments. Instead of a rotating disk electrode a 5 pores per inch (ppi) porous glassy carbon electrode with an estimated area of 13.2 cm^2^ was used as the cathode. µAutolab Potentiostat was used to apply constant potentials between 0.9 to 1.6 V vs. Ag/AgCl for 1 hr. At the end of the experiments the samples were collected for ammonia measurements.

### Flow Cell Experiments

The home-made flow-by electrolysis cell described in Sahin et al was used for flow cell experiments [19]. 80 pores per inch (ppi) glassy carbon electrode were used as the cathode and a dimensionally stable electrode (mesh ID 11873, De Nora) was used as anode. The anode and cathode were separated by a cation exchange membrane purchased from Snow pure (California, USA). Glassy carbon electrodes were obtained from Erg Materials and Aerospace (California, USA) with the following dimensions (0.5 cm×10.0 cm×0.5 cm). Contact to these electrodes was established using Toray paper (Fuel Cell Store) and conductive carbon cement adhesive (SPI Supplies, West Chester, PA). Power supplies (Agilent Technologies and GW Instek Group) were used in constant current experiments to apply 2.46 A which is equivalent to 49.2 mA/cm^2^ based on cross sectional area of the flow cell. A peristaltic pump (Masterflex, Cole Palmer) was used to flow the catholyte and anolyte at a rate of 0.8 ml/s. pH controller purchased from Omega (USA) and was used to keep the pH between 8.4 and 8.6 at the catholyte. Experiments were run until 80% conversion was achieved and samples were collected throughout the experiment for ammonia measurement.

## Supporting Information

Figure S1 **Ammonia removal efficiency and observed biomass yield for continuous flow cultivated** ***Nitrosomonas europaea*** **.**
(TIFF)

Table S1 **Half-cell reactions and thermodynamic cell potential for inorganic electron mediators.**
(DOC)

Table S2 **Summary of biokinetic and stoichiometric parameters describing growth of wild-type** ***Nitrosomonas europaea*** **on electrochemically regenerated media.**
(DOC)

Table S3 **Thermodynamic and biokinetic properties of common inorganic electron mediator couples.**
(DOC)

Table S4 **Physical, thermodynamic and biokinetic properties of ammonia, nitrite, iron (II), hydrogen sulfide and hydrogen.**
(DOC)
