# Supplementary figures and images for: Biomass Production from Electricity Using Ammonia as an Electron Carrier in a Reverse Microbial Fuel Cell
Source: PLoS One. 2012 Sep 19;7(9):e44846. doi: 10.1371/journal.pone.0044846 (PMC3446996; doi:10.1371/journal.pone.0044846)

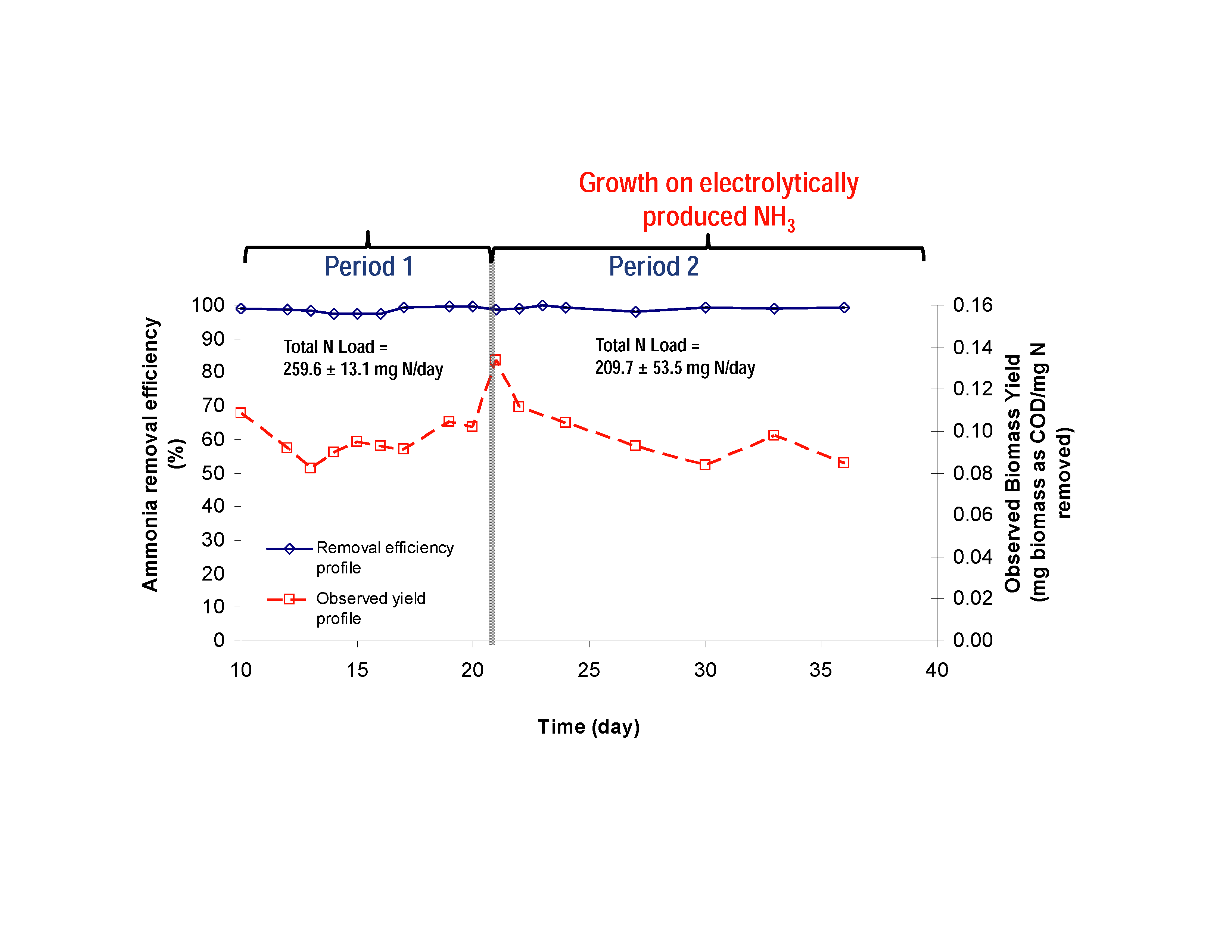

Supplement: Figure S1 — Ammonia removal efficiency and observed biomass yield for continuous flow cultivated Nitrosomonas europaea . (TIFF) [file pone.0044846.s001.tiff]
