# Supplementary material for: Biomass Production from Electricity Using Ammonia as an Electron Carrier in a Reverse Microbial Fuel Cell
Source: PLoS One. 2012 Sep 19;7(9):e44846. doi: 10.1371/journal.pone.0044846 (PMC3446996; doi:10.1371/journal.pone.0044846)
Supplement: Table S1 — Half-cell reactions and thermodynamic cell potential for inorganic electron mediators. (DOC) [file pone.0044846.s002.doc]

**Table S1. Half-cell reactions and thermodynamic cell potential for inorganic electron mediators.**

| **Mediator Couple (Operating pH)** | **Reactions at Anode, Cathode, and Overall Reactions** | **Thermodynamic Cell Potential (V) vs. SHE After Nernst Correction for pH** | **Gibbs Free Energy (kJ/mol donor)^1^** |
| --- | --- | --- | --- |
| NO_2_^-^ ,NH_3_ | Cathode: NO_2_^-^ + 5H_2_O + 6e^-^ ↔ NH_3_ + 7OH^-^ | E= 0.32 |  |
| (pH 7.0) | Anode: 2H_2_O ↔ 4H^+^ + 4e^-^ + O_2_ | E= 0.81 |  |
|  | Overall Reaction: 2NO_2_^-^ + 4H_2_O ↔ 2NH_3_ + 3O_2_ + 2OH^-^ | **E_cell_= -0.49** | -287 |
| NO_3_^-^,NO_2_^-^ | Cathode: NO_3_^-^ + H_2_O + 2e^-^ ↔ NO_2_^-^ + 2OH^-^ | E= 0.41 |  |
| (pH 7.0) | Anode: 2H_2_O ↔ 4H^+^ + 4e^-^ + O_2_ | E= 0.81 |  |
|  | Overall Reaction: 2NO_3_^-^ ↔ 2NO_2_^-^ + O_2_ | **E_cell_= -0.40** | -235 |
| Fe^+3^, Fe^+2^ | Cathode: Fe^+3^ + 1e^-^ ↔ Fe^+2^ | E= 0.77 |  |
| (pH 1.9) | Anode: 2H_2_O ↔ 4H^+^ + 4e^-^ + O_2_ | E= 1.12 |  |
|  | Overall Reaction: 4Fe^+3^ + 2H_2_O ↔ 4Fe^+2^ + 4H^+^ + O_2_ | **E_cell_= -0.35** | -34 |
| SO_4_^-^, H_2_S | Cathode: SO_4_^-2^ + 10H^+^ + 8e^-^ ↔ H_2_S + 4H_2_O | E= -0.21 |  |
| (pH 7.0) | Anode: 2H_2_O ↔ 4H^+^ + 4e^-^ + O_2_ | E= 0.81 |  |
|  | Overall Reaction: H_2_SO_4_ ↔ H_2_S + 2O_2_ | **E_cell_= - 1.02** | -988 |
| H^+^, H_2_ | Cathode: 2H^+^ + 2e^-^ ↔ H_2_ | E= -0.414 |  |
| (pH 7.0) | Anode: 2H_2_O ↔ 4H^+^ + 4e^-^ + O_2_ | E= 0.81 |  |
|  | Overall Reaction: 2H_2_O ↔ 2H_2_ + O_2_ | **E_cell_= - 1.23** | -237 |

^1^Negative sign denotes free energy released from aerobic oxidation of reduced form of mediator
