# Supplementary material for: Biomass Production from Electricity Using Ammonia as an Electron Carrier in a Reverse Microbial Fuel Cell
Source: PLoS One. 2012 Sep 19;7(9):e44846. doi: 10.1371/journal.pone.0044846 (PMC3446996; doi:10.1371/journal.pone.0044846)
Supplement: Table S2 — Summary of biokinetic and stoichiometric parameters describing growth of wild-type Nitrosomonas europaea on electrochemically regenerated media. (DOC) [file pone.0044846.s003.doc]

**Table S2. Summary of biokinetic and stoichiometric parameters describing growth of wild-type *Nitrosomonas europaea* on electrochemically regenerated media.**

|  | **Maximum specific growth rate (µ_max_; 1/day)** | **Free energy efficiency (%)** |
| --- | --- | --- |
| Period 1^a^ | 0.59 ± 0.19 | 5.5 ± 0.5 |
| Period 2^b^ | 0.40 ± 0.11 | 5.9 ± 1.0 |

*^a^n =18, ^b^n=14*

Mean ± 95% confidence interval
