# Supplementary material for: Biomass Production from Electricity Using Ammonia as an Electron Carrier in a Reverse Microbial Fuel Cell
Source: PLoS One. 2012 Sep 19;7(9):e44846. doi: 10.1371/journal.pone.0044846 (PMC3446996; doi:10.1371/journal.pone.0044846)
Supplement: Table S3 — Thermodynamic and biokinetic properties of common inorganic electron mediator couples. (DOC) [file pone.0044846.s004.doc]

**Table S3. Thermodynamic and biokinetic properties of common inorganic electron mediator couples.**

| **Redox Couple** | **Maximum specific growth rate^1^ (µ_max_;1/day)** | **Biomas yield coefficient^2^ (Y;mg biomass produced/mg substrate consumed)** | **pH range^3^** | **Representative biocatalyst considered^3^** |
| --- | --- | --- | --- | --- |
| NH_3_/NO_2_^-^ | 1 | 0.12 | 6 to 8 | *Nitrosomonas europaea* |
| NO_2_^-^/NO_3_^-^ | 0.6 | 0.02 | 6 to 8 | *Nitrobacter winogradskyi* |
| Fe^2+^/Fe^3+^ | 1.4 to 43.2 | 0.02 to 0.28 | 2 to 4 | *Acidithiobacillus ferrooxidans* |
| H_2_S/SO_4_^2-^ | 2.5 to 7.6 | 0.08 to 0.11 | 6 to 8 | *Thiobacillus tepidarius* |
| H_2_/H^+^ | 7.4 to 10.1 | 0.51 to 0.55 | 7 | *Ralstonia eutropha H16* |
| C_6_H_12_O_6_/CO_2_ | 25.2 | 0.36 to 0.60 | 6 to 8 | *Escherichia coli* |

^1^Further details provided in

^2^Biomass is denoted as C_5_H_7_O_2_N; Substrate defined as NH_3_, NO_2_^-^, H_2_S, Fe^2+^, H_2_, C_6_H_12_O_6_

^3^Further details provided in

**References**

1. Madigan MT, Brock TD (2009) Brock biology of microorganisms. San Francisco, CA: Pearson/Benjamin Cummings. xxviii, 1061 p. p.

2. White D (2000) The Physiology and Biochemistry of Prokaryotes. Oxford, United Kingdom: Oxford University Press.

3. Bergey DH, Holt JG (1994) Bergey's manual of determinative bacteriology. Baltimore: Williams & Wilkins. xviii, 787 p. p.

4. Friedrich CG, Rother D, Bardischewsky F, Quentmeier A, Fischer J (2001) Oxidation of reduced inorganic sulfur compounds by bacteria: Emergence of a common mechanism? Applied and Environmental Microbiology 67: 2873-2882.

5. Kanagawa T, Mikami E (1989) Removal of Methanethiol, Dimethyl Sulfide, Dimethyl Disulfide, and Hydrogen-Sulfide from Contaminated Air by Thiobacillus-Thioparus Tk-M. Applied and Environmental Microbiology 55: 555-558.

6. WEF Press (2010) Nutrient Removal: WEF Manual of Practice No. 34. Alexandria McGraw Hill.

7. Wood AP, Kelly DP (1986) Chemolithotrophic Metabolism of the Newly-Isolated Moderately Thermophilic, Obligately Autotrophic Thiobacillus-Tepidarius. Archives of Microbiology 144: 71-77.

8. Mignone C, Donati ER (2004) ATP requirements for growth and maintenance of iron-oxidizing bacteria. Biochemical Engineering Journal 18: 211-216.

9. Nemati M, Harrison STL, Hansford GS, Webb C (1998) Biological oxidation of ferrous sulphate by Thiobacillus ferrooxidans: a review on the kinetic aspects. Biochemical Engineering Journal 1: 171-190.

10. Ishizaki A, Tanaka K (1990) Batch Culture of Alcaligenes-Eutrophus Atcc 17697t Using Recycled Gas Closed-Circuit Culture System. Journal of Fermentation and Bioengineering 69: 170-174.

11. Luli GW, Strohl WR (1990) Comparison of growth, acetate production, and acetate inhibition of Escherichia coli strains in batch and fed-batch fermentations. Appl Environ Microbiol 56: 1004-1011.

12. Siegel RS, Ollis DF (1984) Kinetics of growth of the hydrogen-oxidizing bacterium Alcaligenes eutrophus (ATCC 17707) in chemostat culture. Biotechnol Bioeng 26: 764-770.
