# Supplementary material for: Biomass Production from Electricity Using Ammonia as an Electron Carrier in a Reverse Microbial Fuel Cell
Source: PLoS One. 2012 Sep 19;7(9):e44846. doi: 10.1371/journal.pone.0044846 (PMC3446996; doi:10.1371/journal.pone.0044846)
Supplement: Table S4 — Physical, thermodynamic and biokinetic properties of ammonia, nitrite, iron (II), hydrogen sulfide and hydrogen. (DOC) [file pone.0044846.s005.doc]

**Table S4. Physical, thermodynamic and biokinetic properties of** ammonia, nitrite, iron (II), hydrogen sulfide and hydrogen.

|  | NH_3_ | NO_2_^-^ | Fe^2+^ | H_2_S | H_2_ |
| --- | --- | --- | --- | --- | --- |
| Solubility in water (g/L)^1,2,3^ | 300 | 820 | 570 | 30 | 0.019 |
| Boiling Point (^0^C) ^1^ | -33.3 | 320 | 2861 | -60 | -253 |
| Autoignition temperature (^0^C) ^1^ | 690 | N/A | N/A | 260 | 560 |
| OSHA permissible exposure limit (mg/m^3^)^4^ | 50 | N/A | NR | 14 | NR^5^ |

^1^- Values

^2^- Values are for iron sulfate

^3^ – vol/vol at 15.6^0^C

^4^PEL - OSHA Permissible Exposure Limit (PEL) for general industry

NR- OSHA does not report a PEL; N/A - not applicable

^5^-Can act as asphyxiant when oxygen levels are below 19.5% by volume

**References**

1. Perry DL, Phillips SL (1995) Handbook of inorganic compounds. Boca Raton: CRC Press.

2. Airgas (2010) Hydrogen Sulfide Material Safety Data Sheet Number 001029.

3. Airgas (2010) Hydrogen Material Safety Data Sheet Number 00102.

4. OSHA (2011) Occupational Safety and Health Guidelines.

5. OSHA (2011) Chemical Sampling Information.

6. NIOSH (2011) NIOSH Pocket Guide to Chemical Hazards.
